# Atten-LTC-Enhanced MoE Model for Agent Trajectory Prediction in Autonomous Driving

**DOI:** 10.3390/s26020479

**Published:** 2026-01-11

**Authors:** Shangwu Jiang, Ruochen Wang, Renkai Ding, Qing Ye, Wei Liu

**Affiliations:** 1 School of Automotive and Traffic Engineering, Jiangsu University, Zhenjiang 212013, China; 2112404074@stmail.ujs.edu.cn (S.J.); liuw@ujs.edu.cn (W.L.); 2 Automotive Engineering Research Institute, Jiangsu University, Zhenjiang 212013, China; drk@ujs.edu.cn (R.D.); yeqing0610@163.com (Q.Y.)

**Keywords:** agent trajectory prediction, attention mechanism, liquid time-constant networks, mixture of experts, autonomous driving, sensor data

## Abstract

The development of sensor technology and deep learning has significantly improved the reliability and practicality of automatic driving technology. In an autonomous driving system, agent trajectory prediction is a complex challenge, which includes the understanding of different and unpredictable behavior patterns of various entities, including vehicles, pedestrians, and other traffic participants, among the data collected by sensors. In this paper, we deeply study two kinds of problems: Single-Agent Trajectory Prediction (SATP) and Multi-Agent Trajectory Prediction (MATP). We propose an innovative model, which combines the attention mechanism and integrates the Liquid Time-Constant (LTC) network with spatio-temporal features and the Mixture of Experts (MoE) framework, termed the Atten-LTC-MoE model. The model is general and extensible to support SATP and MATP problems in different autonomous driving environments. In order to improve computational efficiency and prediction accuracy, lane and agent vectorization, spatio-temporal features, agent data fusion, and trajectory endpoint generation technologies are studied. The effectiveness of our method is verified by comprehensive experiments on Argoverse and Interaction datasets. Our proposed model has been superior to the state-of-the-art models in terms of minADE_6_ and minFDE_6_ metrics and has shown significant advantages in the accuracy of agent trajectory prediction and computational performance.

## 1. Introduction

Accurately predicting the driving trajectory of agents in dynamic environments can make agents much safer, efficient, and reliable in the rapidly developing field of autonomous driving [1]. Using advanced predictive analysis technologies and deep learning algorithms, the existing agent trajectory prediction (ATP) system can accurately predict the future trajectory for surrounding agents [2]. It is essential for the decision-making process in autonomous driving, such as path planning [3], collision avoidance [4], and speed regulation [5], which are highly dependent on trajectory prediction results. Taking autonomous vehicles as an example, ATP systems can predict the behavioral intentions of other road users (such as pedestrians, non-motorized vehicles, and other vehicles), dynamically adjust their own driving trajectory, and ultimately achieve higher safety and traffic efficiency navigation in complex traffic scenarios. In addition, ATP also plays a critical role in the fields of traffic management and urban planning, providing data support and decision-making basis for enhancing the overall efficiency of transportation networks by analyzing traffic flow patterns. In addition, ATP is of vital importance in the field of traffic management and urban planning. Through the analysis of traffic flow patterns, it improves the overall efficiency of the traffic network and provides data support and decision-making basis.

Researchers of trajectory prediction generally divide the research in the ATP field into two categories: Single-Agent Trajectory Prediction (SATP) and Multi-Agent Trajectory Prediction (MATP). SATP is generally utilized to predict the future trajectory of a single agent around the target vehicle. This approach is especially suitable for scenarios with sparse traffic flow or the need to handle isolated intelligent agents separately. However, in dense traffic environments, SATP is difficult to fully capture the complex interactive relationships between intelligent agents, thus having certain limitations.

However, MATP extends the prediction dimension to multi-agent collaborative analysis, fully considering the dynamic interactions among agents in the prediction process, and recognizing that the motion state of a single agent will have a significant impact on the driving trajectories of other agents. In complex traffic environments, the interaction between multiple intelligent agents directly determines the traffic flow status and driving safety. Therefore, MATP technology has become the core support for autonomous driving navigation in this scenario. Developing complex MATP models that can accurately capture the interaction mechanisms of intelligent agents is a major research challenge in the current field and a key breakthrough point for fully unleashing the potential of autonomous driving technology applications. The core of MATP lies in the deep integration of advanced computational models and real-time data analysis techniques, which work together to predict the potential future motion states of surrounding intelligent agents. This predictive capability is not only the core basis for autonomous vehicle maneuver control and path planning, but also a key technical support for enhancing system situational awareness in complex traffic environments. With the rapid development of machine learning algorithms, especially in the fields of deep learning and reinforcement learning, the performance of trajectory prediction models has reached unprecedented levels. Meanwhile, the continuous improvement of high-quality sensor data acquisition capabilities has laid a solid foundation for achieving significant technological breakthroughs in this field.

Autonomous driving systems need to process massive amounts of spatial and temporal data to achieve precise trajectory prediction. In this process, autonomous driving systems not only need to take the influence of environmental factors into account, but also need to fully consider the interaction between intelligent agents. The effectiveness of such applications largely depends on whether the underlying prediction algorithms can efficiently capture two key types of information: temporal dependencies (i.e., the historical motion patterns of agents) and spatial relationships (i.e., the interaction relationships among agents). In the research of trajectory prediction technology, the breakthrough studies of Biktairov et al. [6] were highly representative; they introduced a bird’s-eye view perspective and combined it with a CNN algorithm to construct a trajectory distribution model, and successfully achieved accurate characterization of spatial patterns in specific scenes. IntentNet [7], as an extension of this research direction, innovatively used raw sensor data to invert vehicle driving intentions, which can capture subtle behavioral clues in complex interactive scenarios and provide richer information input for the optimization of decision-making. To further overcome the limitations of single data modality, the multimodal framework [8] effectively improves the robustness and accuracy of trajectory prediction by integrating multidimensional features such as vision and motion. Considering the impact of uncertainty factors on prediction results in a dynamic traffic environment, methods like [9] integrate uncertainty estimation into the motion prediction model and provide a risk avoidance basis for the safe navigation of the auto drive system by outputting the probability distribution range of the prediction results. In addition, graph-based neural networks such as LaneRCNN [10], which rely on distributed spatial reasoning capabilities to model spatial correlations between intelligent agents, have shown outstanding performance in precise trajectory prediction tasks, fully verifying the potential application of graph-based models in the field of motion prediction.

Although many researchers have made breakthroughs in trajectory prediction, they still face the problem of computational efficiency in trajectory prediction. The existing methods based on LSTM and Transformer still rely on a large number of parameters to represent complex dependencies, which will incur great computational costs. Improving the parameter efficiency of the trajectory prediction algorithm is the key to realizing the prediction accuracy of the model and improving the calculation feasibility. It can realize real-time operation while ensuring maximum performance and avoiding unnecessary performance degradation.

In this work, we propose a model with low computational cost, which combines the Liquid Time-Constant (LTC) network [11] with the attention mechanism and the Mixture of Experts with Transformer model to provide a low-complexity framework model to improve computational efficiency and prediction accuracy. The main contributions are as follows:

1. A Spatio-Temporal Attention-enhanced encoder–decoder with Liquid Time-Constant is designed. Aiming at the long-term time dependencies of the agent, the dynamic behavior of the agent is extracted from the historical trajectory information, and the spatial interaction of surrounding agents is involved to further improve the prediction accuracy.
2. Improved computational efficiency. Compared with the existing state-of-the-art model, our model can obtain higher prediction accuracy in a smaller parameter scale. The effectiveness of the Atten-LTC-MoE model is verified by a large number of experiments on the Argoverse dataset [12] and in the Interaction dataset [13], which proves that it is suitable for the real-time deployment of single-agent trajectory prediction and multi-agent trajectory prediction.
3. The proposed method in this paper has better expressive and uncertainty control capabilities in modeling multimodal trajectory distribution and dynamic interaction relationships in complex urban intersection scenes by using vectorized data representation, feature fusion, and prediction methods based on multi-input encoders.

The rest of this paper is as follows: Section 2 reviews relevant research works in the field of agent trajectory prediction; Section 3 elaborates on the architecture and technical details of our proposed model; Section 4 presents the experimental settings and results analysis; Section 5 summarizes and prospects the research content of the entire paper.

## 2. Related Work

### 2.1. Agent Trajectory Prediction

Single-agent tracks prediction (SATP) is an essential issue in autonomous driving technology, and its research focus is to predict the future trajectory of a single agent in a complex environment. Although this task may not seem as complex as predicting motion in multi-agent contexts, it also presents its own unique challenges and subtle differences. The core of SATP is to understand and predict the behavior of a single agent, such as a single car, pedestrian, or other traffic participant, in dynamic, unpredictable environments. This prediction is of vital importance for the safety of autonomous vehicles and the trafficability of road traffic, because it directly affects the decision-making processes such as path planning [3], collision avoidance [4], and speed regulation [5]. The complexity of this problem is affected by the historical movement mode, environmental background, and the potential interaction with invisible factors. Researchers strive to improve the accuracy and robustness of these predictions by using advanced machine learning technologies, especially in the field of deep learning and probabilistic modeling.

In the field of single-agent prediction for autonomous driving, some groundbreaking research has made great contributions. Biktairov et al. [6] proposed a method for predicting the motion of a single agent using a bird’s-eye view and a convolutional neural network (CNN, emphasizing the trajectory distribution within unique scenes. As a supplement, IntentNet [7] shifted the focus to directly understand the intention of the agent from the original data of the sensor, and used the deep learning technology to explore the interpretability of complex behavior information. In order to further explore the importance of considering multiple data models, the paper [8] demonstrated the method of integrating different data forms to improve the trajectory prediction accuracy of the monomer. As for the key aspect of uncertainty, the paper [9] proposed a method of motion prediction using uncertainty estimation, which had a much better prediction effect for unpredictable traffic scenes. LaneRCNN [10] provided a graph-centered motion prediction method, which uses distributed representation to predict the trajectory of a single agent. This method shows that graph-based neural networks have great prediction potential for spatial motion reasoning. Based on the above research, many single-agent trajectory prediction technologies have broken through different challenges, and also helped to develop more complex and effective single-agent trajectory prediction models.

**Figure 1:**
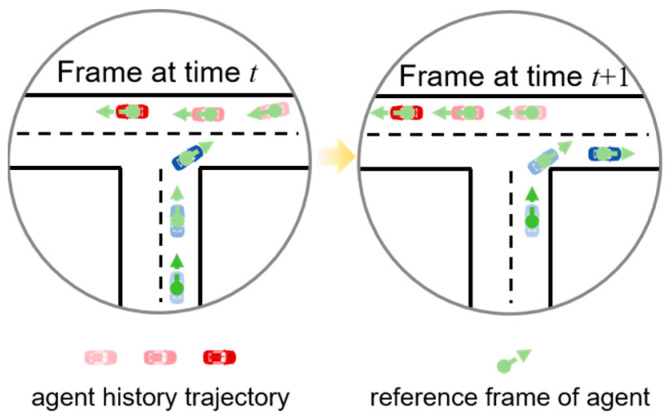
Example of multi-agent trajectory prediction.

On the other hand, Multi-Agent Trajectory Prediction (MATP) emerges as a pivotal and inherently challenging task, as real-world traffic scenarios are characterized by intricate interactions among multiple road agents. The specific prediction method of MATP is shown in Figure 1. Accurately anticipating the future trajectories of all surrounding agents is indispensable for autonomous vehicles (AVs) to make safe, efficient, and context-aware decisions, thereby ensuring smooth navigation in complex traffic ecologies while mitigating collision risks. The success of MATP systems hinges on their capability to capture not only the individual motion patterns of each agent but also the dynamic spatio-temporal dependencies and interactive behaviors that arise from the mutual influences among agents. Recently, a wealth of groundbreaking research has advanced the state-of-the-art in MATP, each addressing distinct facets of the multi-agent interaction problem and collectively shaping a more robust foundation for autonomous navigation. One notable line of work in MATP leverages graph neural networks (GNNs) to explicitly model the interactions among agents. Paper [14] contributed to Multi-Agent Trajectory Prediction (MATP) by proposing a multi-modal Graph Attention Isomorphism Network (GAIN)-based framework, which effectively understood and aggregated long-term interactions across agents while taking model complexity and computational efficiency into account to adapt to real-time application scenarios. Boris Ivanovic et al. [15] proposed Trajectron model that integrated a conditional variational auto encoder (CVAE), LSTM, and a dynamic spatio-temporal graph framework, which can explicitly address the multi-modality, dynamics, and variability of agent behavior, enabling simultaneous prediction of the distribution of future trajectories of multiple agents.

These studies above emphasize the multifaceted nature of MATP in autonomous driving, with each study addressing the unique challenge of capturing the complexity of multi-agent traffic. As autonomous driving technology continues to evolve towards large-scale deployment, there are still some challenges to the insights and methods of these works. The deployment of large models leads to a huge amount of computation within the models, resulting in low computational efficiency. In addition, in dynamic environments, achieving accurate trajectory prediction still faces significant challenges because of complex spatio-temporal correlations between agents. Existing models are often limited by high computational costs and large parameter scales, so it is difficult to deploy them in complex traffic scenes in real time. Therefore, a more lightweight design is urgently needed to meet the real-time requirements of the actual scene. In order to solve the problems above, it is extremely necessary to develop innovative algorithms that can ensure prediction accuracy and have efficient computing capabilities.

### 2.2. Model-Based LSTM and Attention Mechanism in Trajectory Prediction

In the task of autonomous driving, when decisions need to be made, especially when the trajectory of the agent is predicted, spatio-temporal modeling is of great importance, because the agent is affected by the long-term past behavior of surrounding vehicles in time and space in complex traffic scenes. Accurately extracting these temporal and spatial dependencies from historical information can make the model understand the temporal evolution and spatial interaction of vehicle trajectories in complex traffic scenes.

Long Short-Term Memory (LSTM) networks [16] has been widely used in trajectory prediction to simulate long-term dependencies. The model based on LSTM [17,18,19] can extract the continuous motion in the agent trajectory using the encoder–decoder framework. For example, the LG-LSTM framework [20] combined LSTM temporal modeling, GCN local interaction, and attention global fusion to achieve local–global dual-layer interactive modeling, while retaining the temporal advantages of LSTM and the interactive advantages of graph models, significantly improving the prediction accuracy of specific operation trajectories. Similarly, paper [21] proposed the Scene-LSTM model, which extracted scene information through a two-level structure of “scene grid–subgrid”, jointly trained dual LSTM (agent motion LSTM and grid Scene LSTM), filtered effective nonlinear motion information through scene data filters (SDF), and took position offset as input to improve the prediction performance of nonlinear intelligent agent trajectories in static crowded scenes.

Attention mechanism is a powerful approach that focuses on temporal or spatial historical information features to extract spatio-temporal information. Combining these mechanisms can further improve the prediction accuracy of trajectory prediction. The spatio-temporal attention LSTM model [22] skillfully uses spatio-temporal attention to extract the dynamic relationship and historical motion state features between the target vehicle and the surrounding vehicles, so that the model can prioritize the key interactive relationships, thus making the model more interpretable. In addition, in order to enhance the prediction of static environment constrained scenes, the paper [23] adopts dynamic and static context-aware networks to identify the weight of surrounding dynamic vehicles combined with an attention mechanism. It can be seen that the attention-based method can narrow the gap between a single vehicle trajectory and its dependence on surrounding vehicles.

### 2.3. Liquid Time-Constant Networks

The core goal of the trajectory prediction decision scenario is to design an algorithm that can not only reflect the generalization ability by learning the coherent representation of its environment but also provide an interpretable explanation of its dynamic changes. It is found that a single algorithm with only 19 control neurons and 32 encapsulated input features connected to the output through 253 synapses can learn to map high-dimensional inputs to steering instructions [24]. Compared with the large-scale black box learning system, the system shows better generalization, interpretability, and robustness. The neural agent can provide autonomous control ability for the trajectory prediction task.

Surprisingly, even tiny organisms such as *Caenorhabditis elegans* [25] have mastered the abilities of trajectory prediction [26], motion control [27] and navigation [28] by virtue of their near-optimal neural system structure and coordinated neural information processing mechanism. In complex real-world scenes such as autonomous driving, this neural-inspired computing approach is expected to yield AI with more expressive ability. Its model can not only ensure accuracy but also provide interpretability. The structure, connectivity patterns, and information processing mechanisms of the nervous system of *Caenorhabditis elegans* directly guide the design of LTC [24].

The adaptive time constant τ of LTC can dynamically adjust the response speed of the hidden state: τ increases in the constant speed scene, and the long-term motion law is stably retained; τ decreases in the sudden change scenario, and track changes are quickly captured. LTC spreads the gradient through the analytical solution of a linear ordinary differential equation (ODE), without the gradient disappearance problem of traditional RNN, balancing the long-time-dependent modeling and gradient stability.

In this work, in order to build a more accurate and computationally efficient agent trajectory prediction model, this study will combine the attention mechanism and the advanced Liquid Time-Constant (LTC) network [11] to extract and capture the past long-term spatio-temporal historical information. LTC is an innovative type of time continuous recurrent neural network, which is utilized to replace the traditional recurrent network, LSTM, which depends on static parameters to control dynamic characteristics, and is used to improve the modeling ability of complex historical spatio-temporal data and make the predicted trajectory more accurate.

### 2.4. Mixture of Expert Methods

Mixture of experts (MoE) plays a wide role in machine learning, as it can improve the performance and scalability of models. A new sparse MoE architecture [29] has been proposed, which strategically allocates computing resources across different “expert” networks based on input data, providing a new breakthrough for scaled visual models. On the basis of improving parameter efficiency, this method enhances the significant characteristics of large-scale image recognition tasks. The sparse gating mechanism of this method dynamically selects expert subsets for each input, providing a scalable alternative to dense architectures and offering a new understanding of efficient resource allocation in deep learning models. Furthermore, Zhou Y et al. [30] proposed an expert selection routing mechanism to optimize the expert selection process during the learning process. This technology enhances traditional MoE frameworks by allowing for more complex routing algorithms based on input data to select the most relevant experts. Their method not only improves predictive performance but also has a certain interpretability of model decisions.

## 3. Proposed Method

To improve the accuracy of agent trajectory prediction, optimize computational efficiency, and reduce the complexity of the model, this paper constructs the Atten-LTC-enhanced Mixture of Experts (MoE) model (Atten-LTC-MoE) to accurately predict the future trajectory of all agents in complex traffic scenarios. The model description is divided into the following aspects.

### 3.1. Overall Framework

**Figure 2:**
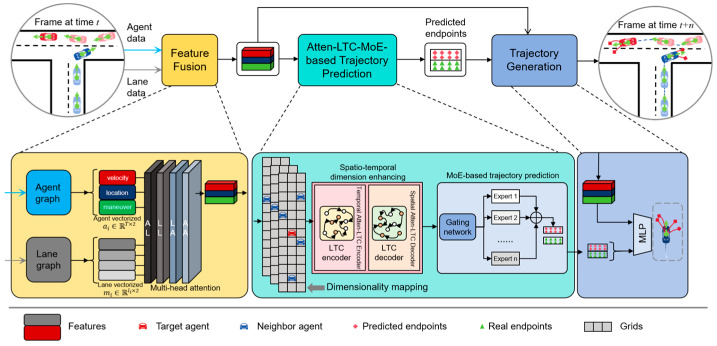
The overall framework of the proposed Atten-LTC-advanced MoE model for trajectory prediction. The proposed method consists of Feature Fusion, Atten-LTC-MoE-based Trajectory Prediction, and Trajectory Generation modules.

The overall framework of the proposed structure is presented in Figure 2, where the entire processing pipeline consists of three essential modules. First, the input data includes the agents’ historical trajectories extracted from high-precision (HD) maps in specific traffic scenarios and the geometric information of surrounding lanes. The input geometric data is usually captured in real-time by sensors installed on the vehicle, such as LiDAR. The feature fusion module first extracts the spatio-temporal features of the agent, as well as key features of motion and lane structure, and then integrates all hidden information into a unified feature representation. The trajectory prediction module based on Atten-LTC-enhanced MoE predicts the key endpoints of the vehicle’s future path based on these fusion features. Finally, the trajectory generation module generates smooth, continuous trajectory curves that conform to physical constraints based on the predicted endpoints. In our proposed model, the LTC unit has stronger temporal and spatial expression ability, which can significantly improve the performance of time series prediction tasks. This model can not only effectively model short-term and long-term dynamic relationships but also fully consider spatial correlation among agents. This innovative fusion enables the model to generate accurate trajectory predictions in dynamic multi-agent traffic scenarios.

### 3.2. Lane and Agent Vectorization Encoding

In the dynamic and complex traffic environment, the data processing efficiency of trajectory prediction is of high importance. The traditional raster representation of maps and agents will produce high-dimensional data, which will lead to the time delay of endpoint processing of the predicted trajectory. In order to improve the efficiency of processing geometric data, we use a vectorized representation [31] to encode geometric data, including time series, lanes, and vehicle tracks. The advantage of vectorization is that it only focuses on the relevant features, which further reduces the computational complexity and data redundancy.

Specifically, we represent the lane information as a set given by $M=\left\{m_{i}\right\}$. Here, the *i*-th lane information is composed of consecutive $l_{i}$ polyline points are encoded into a vector expressed as $m_{i}\in R^{l_{i}\times 2}$, where $l_{i}=\left\{v_{1},v_{2},\ldots ,v_{n}\right\}$, each $v_{i}=\left\{d_{i}^{s},d_{i}^{e},a_{i},j\right\}$ is a vector encoding attributes such as position, heading, or speed, where $d_{i}^{s}$ and $d_{i}^{e}$ denote the coordinates of the starting points and end points of the vector, and $a_{i}$ corresponds to attributes, such as object type, time stamp of track, or road feature type, or speed limit of lane. For each polyline, this work first applies a local GNN [31] to encode the interaction between vectors within the polyline. The polyline is regarded as a fully connected graph, where each node corresponds to a vector $v_{k}$. For each polyline, the local GNN is represented by the message passing update vector:

(1) $$h_{k}^{\left(l+1\right)}=MLP\left(h_{k}^{\left(l\right)}+\sum_{j\in N\left(k\right)}h_{j}^{\left(l\right)}\right)$$

where $h_{k}^{\left(l\right)}$ is the feature of vector *k* in the *l* layer. After the sum operation aggregates the information from adjacent vectors $N\left(k\right)$ to obtain the local polyline coding and further uses the global interaction graph to model the interaction between the agents and the map elements. The nodes in the global graph correspond to the encoded polyline features, and the edges between nodes capture the interaction patterns of spatial and temporal dimensions. Global GNN captures the complex relationship between different polylines through the following formula:

(2) $$h_{i}^{\left(l+1\right)}=MLP\left(h_{i}^{\left(l\right)}+\sum_{j\neq i}h_{j}^{\left(l\right)}\cdot e_{ij}\right)$$

where $e_{ij}$ represents the learning interaction weight between the polyline $l_{i}$ and $l_{j}$, and $h_{i}^{\left(l\right)}$ is the feature vector of polyline *l* in the *i*-th layer. As shown in Figure 2, we construct two independent graph networks—the lane graph and the agent graph—which are, respectively, used for encoding lane and vehicle features. Through these two dedicated graph networks, vehicle trajectory and lane information are efficiently encoded into a vectorization format. The agent feature encoding involves converting the agent trajectories into a vectorized format. The history trajectories of an agent *i* are expressed as $A=\left\{a_{i}\right\}$, where $a_{i}\in R^{T\times 2}$ denotes the 2D history location information of the agent *i* for the previous T time steps in the HD map.

In this work, the lanes in the map are modeled in a vectorization format. The encoding process first converts the input time series or images into tokens into a vector form called embeddings. Consider an input sequence with a length of n, expressed as $\left(x_{1},x_{2},{\ldots ,x}_{n}\right)$, where each $x_{i}$ is a vector in $R^{d_{model}}$. These embeddings are designed to encapsulate the essence of each token and form the basis of the model’s computational processes. In addition, we also use a vectorized graph structure, which is suitable for modeling relationship and structure information. We use the polyline subgraph to encode each scene element.

### 3.3. Feature Fusion for Lane and Agent

Meanwhile, the other critical part is to find an effective approach to model the hidden interaction between different elements in the given input data. The first step in preparing the transaction data for the model involves embedding each transaction $t_{i}$ into a higher-dimensional space, resulting in a sequence of embedded vectors $E=\left\{e_{1},e_{2},\ldots ,e_{n}\right\}$, where each $e_{i}\in R^{d_{model}}$. This embedding process captures the essential features of each transaction, transforming them into a form that is amenable to the subsequent encoding steps in the Transformer architecture. In our proposed method, we adapt a linear layer to encode the input credit card transaction records. This learnable linear layer is crucial for transforming the raw transaction data into a format that is more comprehensible for the model.

**Figure 3:**
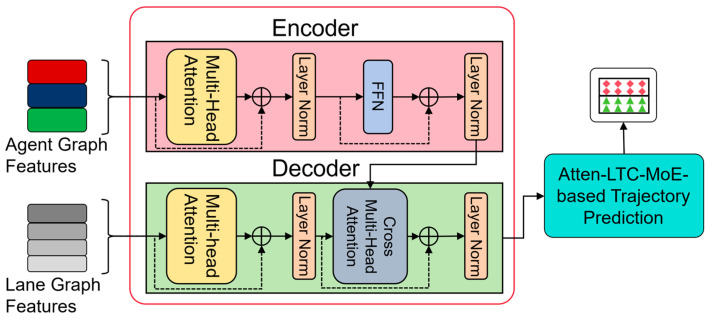
Encoder–decoder architecture and Multi-Head attention (MHA) are used in the feature fusion module.

Transformer architecture includes two main components: encoder and decoder [32], as shown in Figure 3. In order to detect and classify the inputs in this study, the focus is still on the encoder. The encoder operates by obtaining a series of input tags and converting them into a compressed low-dimensional form. Subsequent models use this transformation representation for prediction or classification. A key aspect of the transformer encoder is the integration of the self-attention mechanism. This mechanism helps to capture and process long-distance relationships in input data, so as to generate a more accurate representation of complex inputs.

In the task of marking sequence, the Transformer uses a self-attention mechanism, but lacks the internal method to identify the sequence. Therefore, the position encodings, which refer to Equation (3), are integrated into the input embedding to provide additional information about the position of each token in the sequence:

(3) $$\begin{array}{c} {PE}_{\left(pos,2i\right)}=\sin\frac{pos}{{10,000}^{2i/d_{model}}}\\ {PE}_{\left(pos,2i+1\right)}=\cos\frac{pos}{{10,000}^{2i/d_{model}}} \end{array},$$

Following the operation above, the Transformer encoder applies the self-attention mechanism to these embeddings. This mechanism allows the model to identify and measure the long-term dependencies in the input text by evaluating the relevance of each embedding relative to other embeddings. After the self-attention phase, the encoder uses one or more feed-forward layers on the result representation.

In this context, the input token comprises queries (Q), keys (K), and values (V), each with the dimensionality $d_{model}$. These components are derived by multiplying the input by three learnable matrices $W_{q}$, $W_{k}$, $W_{v}$:

(4) $$Attention\left(Q,K,V\right)=Softmax\left(\frac{QK^{T}}{\sqrt{d_{k}}}\right)V$$

(5) $$Q=W_{q}\cdot x,\;K=W_{k}\cdot x,V=W_{v}\cdot x$$

Here, $d_{k}$ is the hidden dimension, typically equivalent to $d_{model}$. This work utilizes scaled dot-product attention.

**Figure 4:**
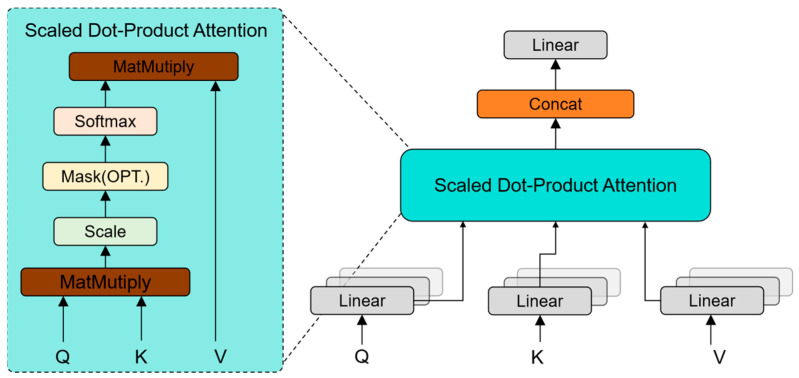
Illustration of the scaled dot-product attention and multi-head attention (MHA) from the Transformer block [32] used in the Feature Fusion Module.

**Multi-head attention mechanism**: This mechanism divides the input embeddings into multiple ‘heads’, applying self-attention independently to each. It allows the model to discern diverse dependencies in the input tokens by weighing the relevance of embeddings within each head. The distinctions between scaled dot-product attention and multi-head attention are showcased in Figure 4, and the output of multi-head attention is formulated as follows:

(6) $$\begin{array}{c} MultiHead\left(Q,K,V\right)=Concat\left({head}_{1},{head}_{2},{\ldots ,head}_{h}\right)W^{O}\\ where\;{head}_{i}=Attention\left(W_{i}^{Q}x,W_{i}^{K}x,W_{i}^{V}x\right) \end{array},$$

The projection matrices are denoted as follows:

(7) $$W_{i}^{Q}\in R^{d_{model}\times d_{q}},W_{i}^{K}\in R^{d_{model}\times d_{k}},W_{i}^{V}\in R^{d_{model}\times d_{v}},W^{O}\in R^{d_{model}\times {hd}_{v}}$$

where ${hd}_{v}=d_{k}$ and h is typically set to 12.

We do not use sinusoidal position coding [32] or absolute position embedding to inject position information. Instead, we add one-dimensional relative position deviation to each attention matrix to consider the relative positions between features in the window. Self-attention in each head with relative bias is calculated as follows:

(8) $$Attention\left(Q,K,V\right)=Softmax\left(\frac{QK^{T}}{\sqrt{d_{k}}}+B\right)V$$

where $B=\left\{b_{i,j}\right\}\in R^{M\times M}$ is a relative position bias, and an element $b_{i,j}={\hat{b}}_{j-1}$ is brought from a learnable bias table $\hat{B}={\left\{{\hat{b}}_{n}\right\}}_{-M+1\leq n\leq M-1}$.

### 3.4. Trajectory Prediction and Generation

Our proposed trajectory prediction module consists of two parts: the endpoint prediction module based on Atten-LTC-MoE, and the trajectory generation module based on MLP. Specific methods are introduced in detail in the following sections.

#### 3.4.1. Atten-LTC-MoE Based Endpoint Prediction

In fact, the output features of agents and lanes in MHA only contain static spatio-temporal relationships, and do not fully capture the dynamic time sequence dependence of agent movement, such as agent acceleration and deceleration trend, long-term following behavior, while dynamic spatial interactions, such as the impact of adjacent agents when changing lanes, are also not contained. On these bases, to enhance the capture of historical information of long-term dependence, this study innovatively utilizes the Atten-LTC unit to establish continuous time and space dynamic features, while making the original highly complex model much more sparse.

**Figure 5:**
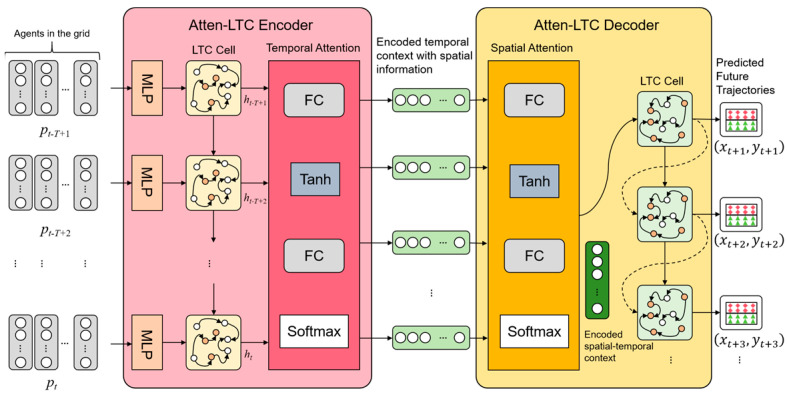
The flow of the spatio–temporal Atten-LTC encoder–decoder module.

Figure 5 introduces the flow of the spatio-temporal attention-enhanced LTC encoder–decoder module in our proposed model. Firstly, in the previous data preprocessing and vectorization module, the model receives the vectorized historical trajectories of the agent in the past t time steps from the 3 × 13 grids. These features are connected in vector form after normalization. In this paper, these characteristics of the *i*-th agent at time t are presented as follows:

(9) $$p_{t}^{i}=\left\{x_{t}^{i},\;y_{t}^{i},\;u_{t}^{i},\;v_{t}^{i}\right\}$$

where $x_{t}^{i}$ and $y_{t}^{i}$ are x coordinate and y coordinate, and $u_{t}^{i}$ defines the agents’ acceleration, while $v_{t}^{i}$ defines the lane change for the *i*-th agent at time *t*. After transforming the motion trajectory of agents into a vectorized data representation, the raw input data is mapped to a high-dimensional space using a multi-layer perceptron (MLP). This potential spatial representation can capture more complex agent behaviors and spatio-temporal dependencies. This work uses an MLP represented by the function ReLU(·) to perform an embedded transformation, which is shown below:

(10) $$r_{t}^{i}=ReLU\left(W_{r}x_{t}^{i}+b_{e}\right)$$

where $W_{r}\in R^{d_{model}\times \;d_{LTC}}$ represents the weight matrix for the fully connected layer. $b_{e}\in R^{d_{model}}$ represents the bias vector. $d_{LTC}$ is the size of the input vectors ($d_{LTC}=4$ for $\left\{x_{t}^{i},y_{t}^{i},u_{t}^{i},v_{t}^{i}\right\}$), $r_{t}^{i}$ is mapped from $d_{model}$ to the hidden dimension of the LTC network $d_{LTC}$ through the linear layer, which is for the *i*-th agent, representing its potential embedding at the time step *t*.

The second step, as shown in Figure 5, then transmits the embedding generated by the MLP layer to the LTC unit, which is to form the time dependency of agents and generate the hidden states $\left\{h_{t-T+1},h_{t-T+2},\ldots {,h}_{t}\right\}$ and $h_{t}\in R^{d_{model}}$ for the historical trajectory of the agents.

LTC model [11] proposed a network hidden state flow composed of linear ordinary differential equations based on the evolution process of CT-RNN [33] hidden state. The specific equation is as follows:

(11) $$\frac{dx\left(t\right)}{dt}=-\left[\frac{1}{\tau }+f\left(x\left(t\right),I\left(t\right),t,\theta \right)\right]x\left(t\right)+f\left(x\left(t\right),I\left(t\right),t,\theta \right)A_{LTC}$$

where $x\left(t\right)$ represents a hidden state, $I\left(t\right)$ represents the input, *t* is the specific time, and the function $f\left(\cdot \right)$ is controlled by a parameter θ. The equation introduces a stable time constant τ that can make the hidden state approach the equilibrium state of the autonomous system. Formula (11) can dynamically adjust the response of the LTC network to historical information according to the time features, so that the LTC model has superior performance in dealing with time-varying dependence.

In terms of specific deployment, the discrete-time approximation method is utilized to achieve a more efficient solution [19]. When the fused ordinary differential equation needs to be solved, the state can be expressed as the following equation:

(12) $$x\left(t+∆t\right)=\frac{x\left(t\right)+∆tf\left(x\left(t\right),I\left(t\right),t,\theta \right)A_{LTC}}{∆t\left(\frac{1}{\tau }+f\left(x\left(t\right),I\left(t\right),t,\theta \right)\right)+1}$$

**Figure 6:**
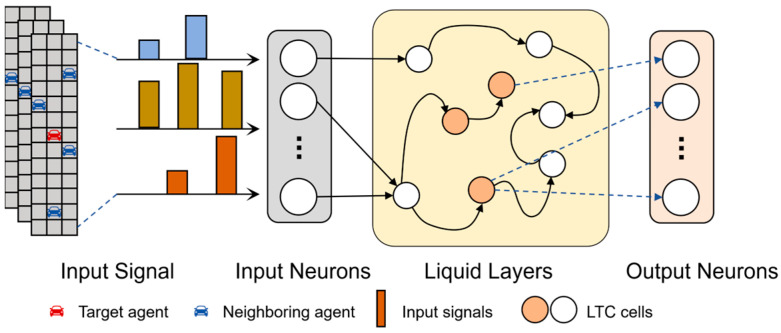
Internal structure of the Liquid Time-Constant (LTC) network.

As shown in Figure 6, the internal structure of each LTC unit is presented. The input neuron first processes the input time series signal, and then inputs the processed time series signal into the liquid layer. Neurons in the liquid layer are interconnected by dynamic pathways, as shown by the arrow. The LTC network has neurons with adaptive time constants, which control the response speed of each neuron to the change in input information. Finally, the output neuron summarizes information after processing.

After generating the hidden states $\left\{h_{t-T+1},h_{t-T+2},\ldots {,h}_{t}\right\}$ of the historical trajectories by LTC unit, the temporal attention module will optimize these hidden states, as shown in Figure 5. The module is composed of fully connected layers (FC), the tanh function, and the softmax function, which can assign attention weight to key time steps. The attention mechanism can give priority to the key time steps by calculating the impact of the historical trajectory on the future motion of the target agent or surrounding agents. Given the hidden state $H_{t}^{k}=\left\{h_{t-T+1}^{k},h_{t-T+2}^{k},\ldots ,h_{t}^{k}\right\}$ of agent k, the time attention weight $A_{t}^{k}$ can be calculated by the following equation:

(13) $$A_{t}^{k}=Softmax\left(W_{t}^{2}tanh\left(W_{t}^{1}H_{t}^{k}\right)\right)$$

where $W_{t}^{i}$ is the learnable weight, and $Softmax\left(\cdot \right)$ is defined as follows:

(14) $$Softmax\left(z_{i}\right)=\frac{e^{z_{i}}}{\sum_{j=1}^{n}e^{z_{i}}}$$

where $z_{i}$ is the *i*-th input for the $Softmax\left(\cdot \right)$ function.

In the following step, encoded contexts with temporal information will be output with spatial information for further decoding:

(15) $$G_{t}^{k}=A_{t}^{k}H_{t}^{k}$$

As we can see in Figure 5, the Atten-LTC decoder module proposed in this study combines the spatial information about the agents with the encoded temporal context through the spatial attention mechanism. This module comprises two key components: the spatial attention module and the LTC decoder. $A_{s}^{k}$ represents the spatial attention weights and is computed as follows:

(16) $$A_{s}^{k}=Softmax\left(W_{s}^{2}tanh\left(W_{s}^{1}G_{t}^{k}\right)\right)$$

where $W_{s}^{i}$ is the learnable weight that controls each agent for the prediction of future trajectories. And the spatio-temporal representation $J_{t}^{k}$ is computed as the following equation:

(17) $$J_{t}^{k}=A_{s}^{k}G_{t}^{k}$$

The generated $J_{t}^{k}$ is then transferred to LTC units, which are endpoints of the rough future trajectories $\left(x_{t+1},y_{t+1}\right),\left(x_{t+2},y_{t+2}\right),\ldots$. This process, through step-by-step decoding, makes sure the seamless integration of spatial and temporal dependencies and generates rough predicted trajectories in a complex multi-agent environment.

To obtain much more accurate predicted trajectories, in this work, we introduce a Mixture of Experts (MoE) [29,30] module to adapt and select the previous rough trajectories. From the previous work [29,30], MoE can more effectively deal with complex, high-dimensional, and nonlinear problems, and obtain excellent predictions by using the analysis and selection of various experts’ work. The basic idea of MoE is to divide the complex problem space into multiple homogeneous regions based on multiple expert networks, so that the system has better adaptability and reflects the universality and robustness.

**Figure 7:**
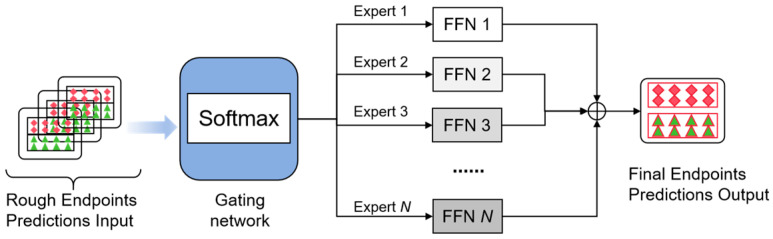
Trajectory prediction using the ensemble Mixture of Experts (MoE).

As shown in Figure 7, the Mixture of Experts (MoE) method in this study employs multiple generic Feedforward Neural Networks (FNNs) as experts and uses linear softmax as the gating network. Similarly to the previously proposed hard MoE, this method achieves sparsity by computing a weighted sum of the outputs from only the top−k experts, rather than a weighted sum of the outputs from all experts. Specifically, within one MoE layer, there exist FNNs, $f_{1},f_{2},\ldots ,f_{n}$, and a gating network $g\left(\cdot \right)$. The gating network is defined as follows:

(18) $$g_{i}\left(x\right)=Softmax\left({top}_{k}\left(W\cdot x+noise\right)\right)$$

where ${top}_{k}$ is a top−k function that only remains the k largest elements in the given vectors. We also add random noise to avoid significant load imbalances. The $Softmax\left(\cdot \right)$ function is computed as follows:

(19) $$Softmax\left(h_{i}\left(x\right)\right)=\frac{exp\left(h_{i}\left(x\right)\right)}{\sum_{j=1}^{k}exp\left(h_{j}\left(x\right)\right)}$$

where $h_{i}\left(x\right)$ is the top−k output of the gating network corresponding to the experts.

The final trajectories output $\hat{y}$ of the MoE model is a weighted sum of expert predictions, where the gating network assigns probabilities $g_{i}\left(x\right)$ to each expert:

(20) $$\hat{y}=g_{i}\left(x\right)f_{i}(x)$$

This modular design significantly improves prediction accuracy and system robustness, especially when dealing with complex and uncertain driving scenarios. In addition, the MoE architecture has the ability to handle heterogeneous data and dynamically adapt to environmental changes, making it particularly suitable for autonomous driving application scenarios. During the training process, the overall loss function of the MoE model combines the prediction results of all experts and is assigned weights by a gating network. The loss function is expressed as the following equation:

(21) $$L=\frac{1}{T}\sum_{t=1}^{T}{\left\|{\hat{y}}_{t}-y_{t}\right\|}^{2}$$

where T represents time steps’ number, ${\hat{y}}_{t}$ denotes the endpoint position at time step t, while $y_{t}$ is the actual endpoint position of the time step t. The model is trained by stochastic gradient descent, while optimizing the parameters of the gating network and various expert models.

#### 3.4.2. Trajectory Generation

The endpoints of the trajectory prediction are essentially a set of discrete coordinate data. In order to generate continuous trajectories that are more consistent with physical constraints, we refer to the method [34] and use the predicted endpoints and their corresponding eigenvectors to convert them into smooth and continuous trajectories. Specifically, the method first uses an MLP layer to predict the position offset of the initial endpoint. Then, an additional MLP layer is used to receive the offset endpoint and its related features and output the final smooth trajectory prediction. At the same time, MLP also generates the corresponding confidence probability distribution for each smooth trajectory.

## 4. Experiments

In this section, we will test the evaluation criterion based on different datasets. The following content analyzes the indicators used and the experimental results.

### 4.1. Experimental Setup

#### 4.1.1. Dataset Specifications

In this work, our evaluation was conducted on the two most popular datasets: the Argoverse dataset [12] for a single-agent prediction task and the Interaction dataset [13] for a multi-agent prediction task.

First of all, we use the Argoverse dataset [12], which has a numerous and diverse dataset, including 323,557 individual scenes, and is a well-known benchmark in the field of single-agent trajectories prediction. In addition, the Interaction dataset [13] is used as the evaluation dataset for multi-agent trajectory prediction. The Interaction dataset contains 62,022 scenarios, and each scenario contains up to 40 agents. It has the future movement of all agents in a given scene and can provide a more complex and diverse multi-agent prediction environment. The above two datasets are randomly scrambled and uniformly divided into the training set, verification set, and test set according to the ratio of 7:1:2.

#### 4.1.2. Evaluation Metrics

Similarly to previous works [34,35], our work utilizes several standard metrics for evaluation, including the minimum Average Displacement Error (${mADE}_{k}$), minimum Final Displacement Error (${mFDE}_{k}$), Miss Rate (${MR}_{k}$), and Brier minimum Final Displacement Error ($brier-{mFDE}_{k}$). These error metrics are calculated based on the trajectory with the closest endpoint to the ground truth over k trajectory predictions. These metrics measure the prediction quality from different angles. The ${mADE}_{k}$ metric measures the average L2 distance between the entire prediction trajectory and the corresponding ground-truth results:

(22) $${mADE}_{k}=\underset{i\in \left\{1,\ldots ,k\right\}}{\min}\left(\frac{1}{T}\sum_{t=1}^{T}{\left\|{\hat{y}}_{i,t}-y_{t}\right\|}_{2}\right)$$

The ${mFDE}_{k}$ metric measures the distance between the predicted endpoints and the ground-truth endpoints:

(23) $${mFDE}_{k}=\underset{i\in \left\{1,\ldots ,k\right\}}{\min}{\left\|{\hat{y}}_{i,T}-y_{T}\right\|}_{2}$$

${MR}_{k}$ is the ratio of scenes where ${mFDE}_{k}$ is larger than 2 m. The $brier-{mFDE}_{k}$ metric is calculated as follows:

(24) $$brier-{mFDE}_{k}={\left(1-p\right)}^{2}+{mFDE}_{k}$$

where p is the probability predicted for the trajectory.

#### 4.1.3. Model Configurations and Training

In this study, a network structure with L = 3 layers of depth was adopted for polyline subgraphs and interactive modeling. The model training process consists of 36 complete epochs, with a batch size set to 256 to balance computational efficiency and memory usage. The Atten-LTC-MoE-based trajectory prediction module is configured with a total of eight expert networks and dynamically selects the top−k = 2 most relevant expert combination prediction results during the inference process. For optimization, we employed the Adam optimizer and set different initial learning rates for different datasets: the Argoverse dataset uses a learning rate of 1 × 10^−4^, while the Interaction dataset uses a learning rate of 2 × 10^−4^ to adapt to more complex multi-vehicle interactions. All experiments were conducted on RTX 4090 GPU servers equipped with 64 GB of memory, ensuring efficient execution of large-scale training.

Referring to the method in this work [34], our proposed method reduced the learning rate to 0.15 times the original value at the 70th and 90th nodes of the training process to promote model convergence to a better solution. In terms of data preprocessing, we generated detailed lane vector representations for roads within 50 m of any agent. To enhance the generalization ability of the model, various data augmentation techniques were implemented, including random scaling within the range of [0.75, 1.25], as well as applying a 0.15 probability random agent dropout strategy for Argoverse and Interaction experiments, simulating sensor occlusion and detection uncertainty. For the reference coordinate system setting, this paper performs scene translation and rotation processing relative to the target agent in the reference frame centered on the agent; Conversely, in the scene-centric frames orientation, the coordinate system direction was determined based on the average position of all relevant agents, providing a more global perspective.

### 4.2. Ablation Study

**Figure 8:**
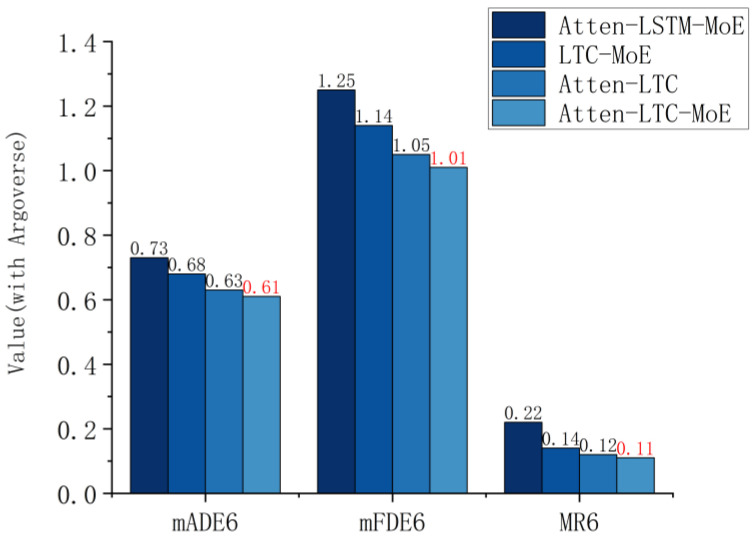
Comparison of Atten-LTC-MoE framework core module ablation experiment on the Argoverse dataset.

In order to quantify the independent contributions of the attention mechanism, LTC unit, and MoE in the Atten-LTC-MoE framework, three sets of model variants were designed and compared in terms of their predictive performance on the Argoverse dataset. The results are shown in Figure 8.

As shown in Figure 8, the ${mADE}_{6}$ of Atten-LSTM-MoE reached 0.73, significantly higher than other variants; the ${mADE}_{6}$ of LTC-MoE (0.68) and Atten-LTC (0.63) decrease sequentially, while the ${mADE}_{6}$ of our model is only 0.61. This result indicates that replacing LTC with LSTM significantly increases the displacement error of short-term trajectories, verifying that LTC’s adaptive time constant is more suitable for dynamic motion patterns in traffic scenarios. The LTC-MoE error of removing spatial attention is higher than Atten-LTC, indicating that spatial attention can effectively integrate the heterogeneous features of agents and lanes, and improve short-term prediction accuracy.

It can be seen that the ${mFDE}_{6}$ of Atten-LSTM-MoE is 1.25, far higher than other models, while the long-term errors of LTC-MoE (1.14) and Atten-LTC (1.05) gradually decrease, and the ${mFDE}_{6}$ of our model is only 1.01. This trend further highlights the advantages of LTC: the static gating mechanism of traditional LSTM is difficult to capture the dependency relationship of long-term trajectories, while LTC avoids the problem of vanishing gradients in long sequences through the analytical gradient propagation of linear ODE, greatly reducing the long-term endpoint error. At the same time, the lack of attention weakens the modeling ability of multi-agent interaction, resulting in long-term errors of LTC-MoE being higher than Atten LTC.

The ${MR}_{6}$ of Atten-LSTM-MoE (0.22) is the highest among all methods, while the failure rates of LTC-MoE (0.14) and Atten-LTC (0.12) decrease sequentially. The ${MR}_{6}$ of our model is only 0.11. The change in failure rate is consistent with the error index; the LTC units can significantly improve the stability of trajectory prediction, while the synergistic effect of attention and MoE further reduces the risk of prediction failure, making the robustness of the model optimal.

**Figure 9:**
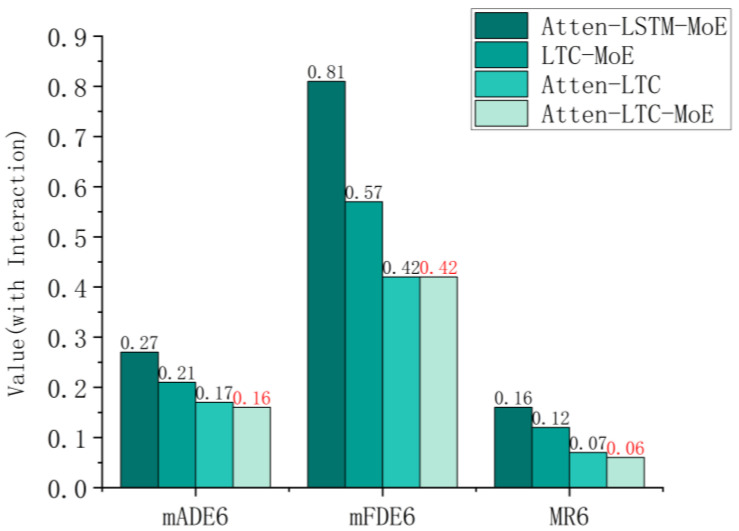
Comparison of Atten-LTC-MoE framework core module ablation experiment on the Interaction dataset.

The Interaction dataset focuses on multi-agent intensive interaction scenarios, which can better validate the modeling ability of the model for complex dynamic interactions. As shown in Figure 9, the ${mADE}_{6}$ of Atten-LSTM-MoE reached 0.27, significantly higher than LTC-MoE (0.21), Atten-LTC (0.17), and our model (0.16). In multi-agent scenarios, vehicle motion is more affected by interference from adjacent agents, while LTC’s adaptive time constant can quickly capture trajectory changes caused by interactions, and spatial attention can encode the relative positional relationships between agents. The combination of the two modules reduces the short-term interaction prediction error of the original model by 40.7% compared to Atten-LSTM-MoE, reflecting the adaptability of the core module to complex dynamic interactions.

The ${mFDE}_{6}$ of Atten-LSTM-MoE is 0.81, which is nearly twice that of our model (0.42). The long-term trajectory of multi-agent systems relies on the dynamic evolution of interaction strategies, such as collaborative lane changing and competition for lanes. The long-term gradient stability of LTC avoids the gradient vanishing problem of traditional LSTM, while the dynamic expert selection of MoE can adapt to different interaction modes. The synergy of the two improves the long-term prediction accuracy of the original model by 48.1% compared to Atten-LSTM-MoE, highlighting the advantages of the framework in long-term interaction modeling.

Extreme interactions in multi-agent scenarios can easily lead to trajectory prediction failure. Therefore, the ${MR}_{6}$ of Atten-LSTM-MoE is 0.16, while our model is only 0.06, reducing the failure rate by 62.5%. The synergistic effect of attention and LTC enhances the robustness of the model to abnormal interactions. Combined with MoE’s sparse inference, it further reduces the risk of failure and significantly improves the robustness of the original model in complex scenarios.

### 4.3. Results and Analysis

#### 4.3.1. Comparison with State-of-the-Art Models

**Figure 10:**
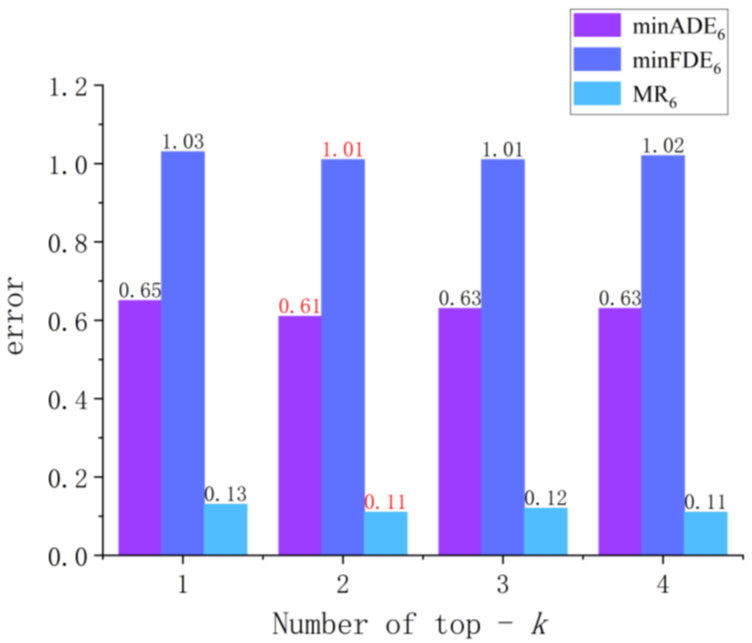
Trajectory prediction using the ensemble Mixture of Experts (MoE). The influence of the number of top−k expert selections on trajectory prediction metrics (${mADE}_{6}$, ${mFDE}_{6}$, ${MR}_{6}$) in the Atten-LTC-MoE framework.

**Parameter test**: as shown in Figure 10 and Table 1, it is clearly presented that the impact of the number of active experts (top−k = 1~4) in the Atten-LTC-enhanced MoE-based module on the three key metrics on the Argoverse dataset. From the data trend, we can see that (1) ${mFDE}_{6}$ as the endpoint error index, it always maintains the highest value (1.01~1.03), indicating that it is difficult to predict the trajectory endpoint; (2) the overall error of ${mADE}_{6}$ is low (0.61~0.65), and it reaches the optimal value when the top−k = 2, indicating that increasing the number of experts moderately helps to improve the overall accuracy of the trajectory predictions; (3) ${MR}_{6}$ is the most stable (0.11~0.13) as the conflict rate metric, which verifies the robustness of the MoE module. It is worth noting that when top−k increases from 1 to 2, all metrics are improved, but the continued increase in the number of experts will lead to fluctuations in some metrics, which provides an important basis for model parameter optimization: in order to achieve the best balance between calculation efficiency and prediction accuracy, top−k = 2 is selected in this work.

**Figure 11:**
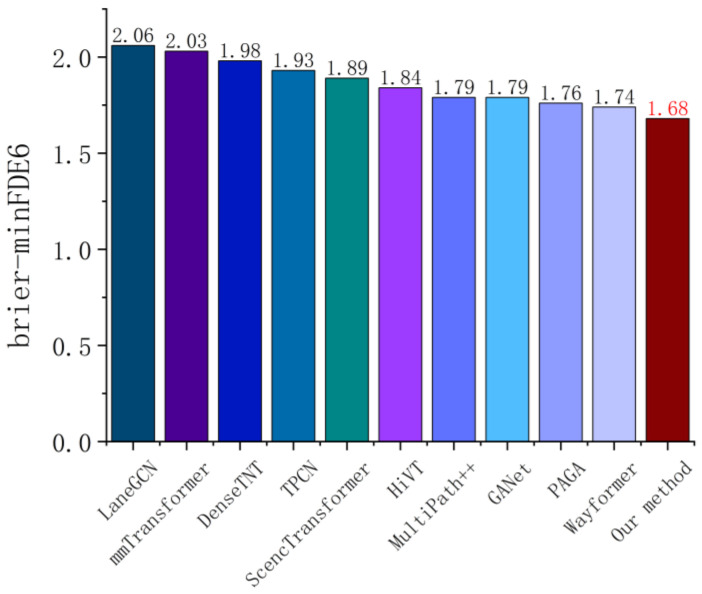
${brier-mFDE}_{6}$ error metric comparison for single-agent trajectory prediction on the Argoverse dataset [12].

**Figure 12:**
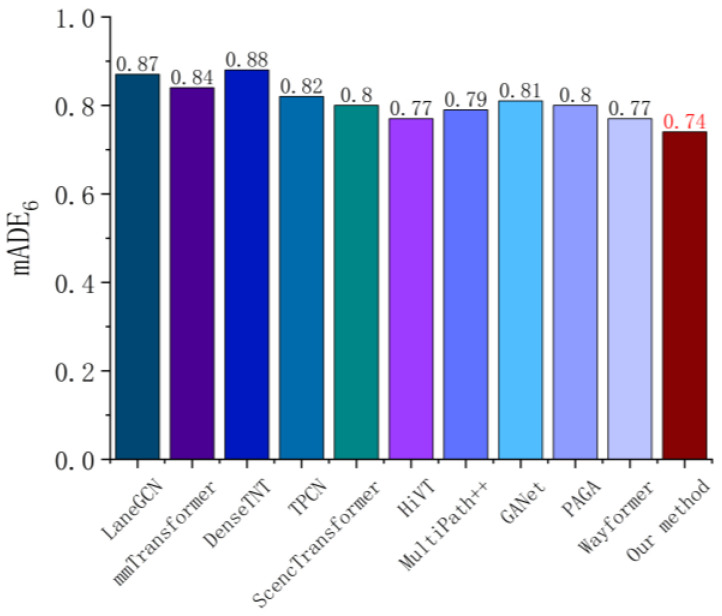
${mADE}_{6}\;$ error metric comparison for single-agent trajectory prediction on the Argoverse dataset [12].

**Figure 13:**
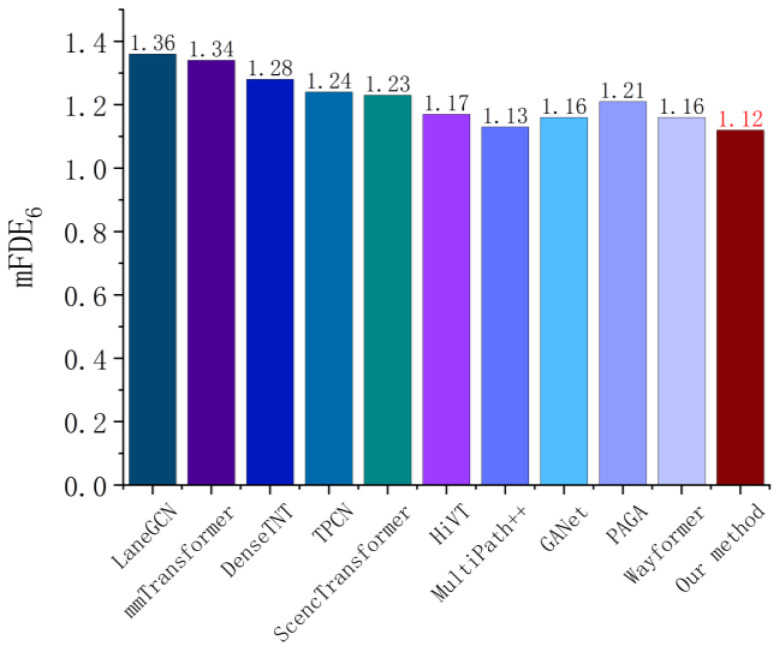
${mFDE}_{6}\;$ error metric comparison for single-agent trajectory prediction on the Argoverse dataset [12].

**Figure 14:**
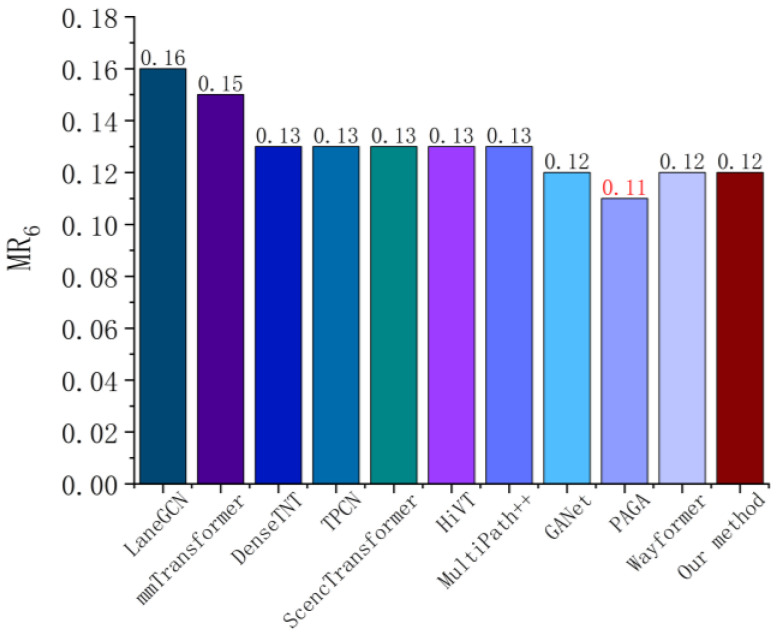
${MR}_{6}$ error metric comparison for single-agent trajectory prediction on the Argoverse dataset [12].

**Compared with the state-of-the-art model**: We first evaluate the single-agent trajectory prediction task, and predict data in Argoverse [12] compared with several of the most state-of-the-art baseline models. Figure 11, Figure 12, Figure 13 and Figure 14 summarize the performance comparison of different models (${brier-mFDE}_{6}$, ${mADE}_{6}$, ${mFDE}_{6}$ and ${MR}_{6}$ as evaluation metrics) for fair comparison. The compared models considered in this test include LaneGCN [36], mmTransformer [37], DenseTNT [38], TPCN [39], SceneTransformer [40], HiVT [41], MultiPath++ [42], GANet [43], PAGA [44], and Wayformer [35]. It should be noted that the results shown in this paper do not use the integration method, except for MultiPath++ (only the integration results are shown).

As can be seen from Figure 11, the method proposed in this paper achieves the optimal result (1.68) on the ${brier-mFDE}_{6}$ metric on the Argoverse dataset, which is superior to the existing popular trajectory prediction algorithms, such as LaneGCN (2.06), DenseTNT (1.98), and Wayformer (1.74). As the model evolves from the traditional Graph Convolution Network structure to the Transformer and Mixture of Experts (MoE) mechanism, the prediction error metrics present a downward trend gradually. This indicates that our method has better expression ability and uncertainty control ability in modeling multimodal trajectory distribution and dynamic interaction in a complex urban intersection scene.

As shown in Figure 12, the ${mADE}_{6}$ of this method on the Argoverse dataset reaches 0.74, which is better than all the models compared, including LaneGCN (0.87), DenseTNT (0.88), HiVT (0.77), and Wayformer (0.77). The overall trend shows that with the model from the traditional Convolution Network to the introduction of the Transformer structure and the fusion of the Mixture of Experts (MoE) mechanism, the trajectory prediction has achieved continuous optimization in the overall average displacement error. The vectorized feature extraction, temporal and spatial attention fusion, and dynamic expert selection strategies used in this paper can better model the complex interaction relationship and multimodal trajectory distribution, thus effectively reducing the overall deviation between the predicted trajectory and the real trajectory.

Figure 13 shows the ${mFDE}_{6}$ error performance of different models on the Argoverse dataset. The method proposed in this paper achieves the minimum error value of 1.12, which is better than Wayformer (1.16), GANet (1.16), MultiPath++ (1.13), and other existing advanced methods. The overall trend shows that, based on the Atten-LTC-advanced MoE mechanism, the model can dynamically select the prediction module according to different driving intentions and effectively improve the prediction accuracy of the end position of the future trajectory, combined with high-precision map vectorized coding. Since ${mFDE}_{6}$ emphasizes the prediction accuracy of the final time, the results further verify the ability of this method to generate a reasonable and coherent trajectory in complex traffic scenes, reflecting the comprehensive advantages of long-term dependence modeling and terminal optimization.

Figure 14 indicates that the comparison of various methods on ${MR}_{6}$ metric. Our method achieves a low error rate of 0.12, which is equal to or slightly superior to the state-of-the-art methods such as GANet and Wayformer, and is better than early methods such as LaneGCN (0.16) and mmTransformer (0.15). It reflects whether there is a large bias between the predicted trajectories and the real trajectories, especially for the safety of autonomous driving decision-making. The multimodal trajectory generation and spatio-temporal interactive modeling mechanism proposed in this paper effectively reduces the frequency of extreme trajectory deviation and improves the robustness and security ability of the system in a complex dynamic environment.

In addition, to verify the balance between accuracy and efficiency of the Atten-LTC-MoE framework, this section tested the core efficiency indicators (parameter count, floating-point operation count, inference delay, batch speed) of mainstream trajectory prediction models based on RTX 4090 GPU (batch size = 256). The results are shown in Table 2.

As shown in Table 2, the parameter count (8.77 M), MFLOPs (12.36), per-sample delay (2.1 ms), and FPS (1190) of our method proposed in this article are all optimal among the benchmark models compared. The advantage comes from the lightweight design of the framework, where the LTC unit adopts a sparse linear ODE structure instead of redundant gate control weights. At the same time, a top-k (k = 2) expert selection strategy is adopted in the MoE module, activating only two experts and avoiding parameter redundancy caused by full expert activation. It means that our method has the ability to balance accuracy and efficiency and is suitable for trajectory prediction needs in large-scale traffic scenarios.

On the other hand, in the more challenging task of multi-agent trajectory prediction, our proposed method is also compared with the state-of-the-art baseline models. Table 3 presents the accuracy comparison of multi-agent trajectory prediction tasks using the Interaction dataset. The comparison still focuses on three key metrics: ${mADE}_{6}$, ${mFDE}_{6}$ and ${MR}_{6}$. Our proposed method performs well in all available metrics. Specifically, it achieves the lowest ${mADE}_{6}$ (0.16), indicating that when considering the first six predicted trajectories, the average displacement error of all time steps is the smallest. Compared with SceneTransformer [40] and Thomas [45], this is a significant improvement, and its ${mADE}_{6}$ is 0.26, respectively. In ${mFDE}_{6}$, the proposed method also performs well, and the final displacement error is 0.42, which is better than SceneTransformer (0.47), ITRA [46] (0.49) and DenseTNT (0.67), indicating higher accuracy in the final prediction step. For the miss-detection rate ${MR}_{6}$, our proposed method provides a competitive miss detection rate of 0.06, which is slightly higher than that of SceneTransformer and THOMAS (0.05). The ${MR}_{6}$ of our proposed methods is better than that of GOHOME [47]. It shows that the method proposed in this work can minimize the number of missed detections while maintaining the superior accuracy of other metrics. Generally, the proposed method shows significant improvement in the accuracy of average and final displacement, while maintaining a low rate of missed detection, making it the most reliable multi-agent prediction method in the dataset.

#### 4.3.2. Qualitative Analysis

**Figure 15:**
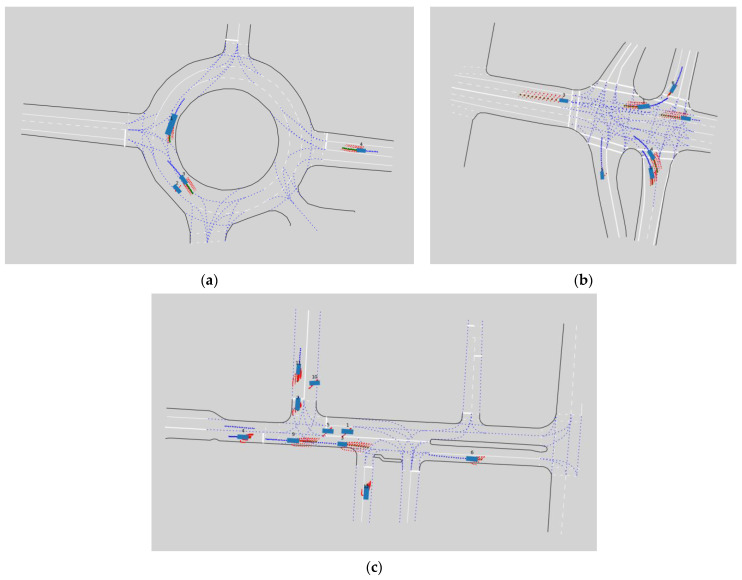
Visualization of multi-agent trajectory prediction results on the Interaction dataset [13]: (**a**) scene CHN roundabout; (**b**) scene USA intersection 1; and (**c**) scene USA intersection 2.

To gain a deeper understanding of the advantages and characteristics of the model proposed in this article, multiple typical scenarios were randomly selected from experimental data for detailed visualization analysis. Figure 15a–c shows the visualization results of multi-agent trajectory prediction for three different complex scenarios on the Interaction dataset [13]. These scenes represent complex road layouts commonly seen in the real world, where multiple agents interact in different traffic environments. The experimental samples in this article cover various typical road environments, including roundabouts and standard intersections.

In these visualizations, the red dots represent the possible trajectories predicted by the model, while the green curves represent the actual observed trajectories. From the visualization results, it can be clearly observed that the model proposed in this paper can generate high-quality multi-modal predictions, fully considering various possible motion paths under the current state of the vehicle and environmental constraints. The predicted trajectory strictly follows the lane structure and traffic rules, indicating that the model effectively integrates map features, such as road layout and lane lines, when predicting future movements, which is crucial for safe and reliable trajectory prediction in the autonomous driving system.

The roundabout scene in Figure 15a particularly demonstrates the model’s ability to handle complex circular road structures, and the predicted trajectory accurately reflects the turning behavior and speed changes in agents in the roundabout. The intersection scenarios in Figure 15b,c demonstrate the reliability of the model in predicting various behavioral patterns such as straight driving and turning. It is worth noting that the trajectories generated by the model not only approach the real trajectory in spatial position but also exhibit high consistency in velocity and acceleration characteristics, which is particularly important for safety planning and risk assessment in autonomous driving.

## 5. Conclusions

In this work, we combine the attention mechanism with LTC and the MoE model to build an extensible framework for trajectory prediction in single-agent or multi-agent complex traffic environments. Firstly, the Transformer model with an attention mechanism is used to fuse the feature vectors extracted from the lane graph and agent graph. Then, the fused feature vectors are mapped to the grids and input into the trajectory prediction module based on Atten-LTC-MoE, and then input into the trajectory generation module to gain the predicted trajectory of the target agent. We have conducted benchmark tests on two popular datasets, Argoverse and the Interaction dataset. The experimental results show that the model based on Atten-LTC-MoE is effective in solving the SATP and MATP tasks for autonomous driving. The LTC unit ensures the sparsity of our proposed model and the fusion attribute of space-time features. The prediction based on the MoE method improves the accuracy and efficiency of trajectory prediction. Future work could explore how to further enhance scalability and real-time processing capability, and reduce the scale of the expert network combined with knowledge distillation technology, consolidating the role of MATP in the technological progress of autonomous vehicles.

## Figures and Tables

**Table 1:** Performance analysis of the impact of the number of activated experts in the Atten-LTC-advanced MoE-based trajectory prediction module on the Argoverse dataset [12].

Top-*k*	minADE_6_	minFDE_6_	MR_6_
1	0.65	1.03	0.13
2	0.61	1.01	0.11
3	0.63	1.01	0.12
4	0.63	1.02	0.11

**Table 2:** Comparison of computational efficiency indicators with baseline prediction models at batch size = 256.

Models	Parameter (M)	MFLOPs	Per-Sample Latency (ms)	FPS
DenseTNT [38]	12.93	21.44	5.1	610
LaneGCN [36]	10.24	18.52	3.4	735
Wayformer [35]	15.69	27.81	5.2	481
Our method	8.77	12.36	3.1	1190

**Table 3:** Performance analysis of the impact of the number of activated experts in the Atten-LTC-advanced MoE-based trajectory prediction module on the Interaction dataset [13].

Models	minADE_6_	minFDE_6_	MR_6_
DenseTNT [38]	0.21	0.67	-
SceneTransformer [40]	0.26	0.47	0.05
ITRA [46]	0.17	0.49	-
GOHOME [47]	-	0.45	0.07
THOMAS [45]	0.26	0.46	0.05
Our method	0.16	0.42	0.06

## Data Availability

Publicly available datasets were used in this study. The Argoverse v1.1 dataset can be found at https://s3.amazonaws.com/argoverse/datasets/av1.1/tars/hd_maps.tar.gz (accessed on 27 September 2025), and the Interaction dataset can be found at INTERACTION Dataset GitHub https://github.com/interaction-dataset (accessed on 27 September 2025).
